# Continuous Decline in Myocardial Infarction and Heart Failure Hospitalizations during the First 12 Months of the COVID-19 Pandemic in Israel

**DOI:** 10.3390/jcm11061577

**Published:** 2022-03-13

**Authors:** Gil Lavie, Yael Wolff Sagy, Moshe Hoshen, Walid Saliba, Moshe Y. Flugelman

**Affiliations:** 1 Branch of Planning and Strategy, Clalit Health Services, Tel Aviv 6209804, Israel; yaelwo@clalit.org.il (Y.W.S.); mbhoshen@gmail.com (M.H.); 2 Ruth and Bruce Rappaport Faculty of Medicine, Technion—Israel Institute of Technology, Haifa 31096, Israel; saliba_wa@clalit.org.il (W.S.); myf@technion.ac.il (M.Y.F.); 3 Department of Community Medicine and Epidemiology, Lady Davis Carmel Medical Center, Haifa 3436212, Israel; 4 Department of Cardiovascular Medicine, Lady Davis Carmel Medical Center, Haifa 3436212, Israel

**Keywords:** COVID-19 pandemic, acute myocardial infarction, congestive heart failure, cardiovascular hospitalizations

## Abstract

Background: A decline in cardiovascular hospitalizations was observed during the initial phases of the COVID-19 pandemic. We examine the continuous effect of the COVID-19 pandemic in reducing cardiovascular hospitalization and associated mortality rates during the first year of the pandemic in Israel. Methods: We conduct a retrospective cohort study using the data of Clalit Health Services, the largest healthcare organization in Israel. We divide the Corona year into six periods (three lockdowns and three post-lockdowns) and compare the incidence rates of cardiovascular hospitalizations and 30-day all-cause mortality during each period to the previous three years. Results: The number of non-STEMI hospitalizations during the first year of the pandemic was 13.7% lower than the average of the previous three years (95% CI 11–17%); STEMI hospitalizations were 15.7% lower (95% CI 13–19%); CHF (Congestive heart failure) hospitalizations were 23.9% lower (95%, CI 21–27%). No significant differences in 30-day all-cause mortality rates were observed among AMI (acute myocardial infarction) patients during most of the periods, whereas the annual 30-day all-cause mortality rate among CHF patients was 23% higher. Conclusions: AMI and CHF hospitalizations were significantly lower during the first year of the pandemic relative to 2017–9. Mortality rates were higher in the case of CHF patients but not in the case of AMI patients, possibly due to a change in the clinical acuity of patients arriving at the hospitals. We conclude that targeted public health messaging should be implemented together with proactive monitoring, in order to identify residual disability in patients who may have received non-optimal treatment during the pandemic.

## 1. Introduction

A significant decline in hospitalization for acute myocardial infarction (AMI) and congestive heart failure (CHF), which can be attributed to social containment mandates, was reported worldwide during the initial phases of the COVID-19 pandemic [1,2,3,4,5,6]. This reduction may have had significant effects on public health, with respect to both out-of-hospital mortality and long-term complications of myocardial infarction, such as fatal arrhythmia and disabling heart failure [7]. The evidence from various other studies for the continuing effect of the pandemic on hospitalization trends is mixed [8,9,10]. In this study, we examine the impact of the COVID-19 pandemic on AMI and CHF hospitalization rates and on 30-day all-cause mortality rates in Israel during the first 12 months of the pandemic, relative to the previous three years.

## 2. Methods

### 2.1. Study Design and Population

A retrospective cohort study was carried out of all AMI and CHF hospitalizations among Clalit Health Services (CHS) members aged 18 and older, during the period 14 March 2017–13 March 2021. CHS is the largest HMO in Israel (where health insurance is universal and mandatory), with a membership of 4.8 million (accounting for 52% of the population). CHS maintains a central computerized database that provides nearly complete digital records for all its members. Furthermore, all hospitals in Israel are required to report patient diagnoses to the payer HMO. Thus, the dataset is virtually complete for the CHS share of the market, which can be viewed as representative of the entire population.

### 2.2. Study Variables

AMI cases were divided into STEMI (ST elevation myocardial infarction) (ICD 9 codes: 4100x, 4101x, 4102x, 4103x, 4104x, 4105x, 4106x, 4108x) and non-STEMI (ICD 9 codes: 4107x, 4109x) while CHF cases were identified as ICD-9 code 428x. The dataset also includes the demographic characteristics of all CHS members aged 18 years and older, including sex, date of birth (used to calculate age, which was subsequently categorized into 60+ and under 60), ethnicity (general, ultra-Orthodox Jewish or Arab, according to the ethnic classification of the member’s primary clinic), and background diagnoses, which were used to calculate the Charlson comorbidity index (CCI; categorized up to 5 and above 5), a measure of comorbidity load.

### 2.3. Comparison Periods

A year for the purpose of the study was defined as beginning on 14 March and ending on 13 March, such that the sample period is from 2017–2018 to 2020–2021, where 2020–21 is the “Corona year”. Incidence rates were calculated at the weekly level. The comparison periods were defined according to the three lockdowns as follows (according to calendar weeks): first lockdown (week 10–15); post-first lockdown (week 16–36); second lockdown (week 37–41); post-second lockdown (week 42–51); third lockdown (week 52–6); and post-third lockdown (week 7–10). (Note that week 52 and week 1 of the following year are partial weeks.)

### 2.4. Data Analysis

Mean weekly incidence rates of hospitalization for STEMI, non-STEMI and CHF were calculated per 1000 person-years. The rates for the Corona year were then compared to the average of the three baseline years for each period. Case fatality rates (CFR), defined as the proportion of all-cause death within 30 days of hospital admission (whether in- or out-of-hospital), were calculated for each period, and the OR for 30-day mortality in the Corona year relative to previous years was estimated using generalized estimating equations (GEE) in a logistic regression, adjusting for age, sex and CCI. All calculations were performed using R software version 4.1.1, together with two basic packages: “RODBC” for data extraction from the data warehouse and “gee”. Significance was set at *p* < 0.05. In view of the descriptive nature of the analysis, no adjustment was made for multiple comparisons. The CHS Institutional Ethics Board approved the study with a waiver of informed consent.

## 3. Results

The breakdown of total cases between the three baseline years and the Corona year is as follows: STEMI—9562 patients (yearly average of 3187) vs. 2758 patients; non-STEMI—22,060 patients (yearly average of 7353) vs. 6509 patients; and CHF—108,856 patients (yearly average of 36,285) vs. 28,352 patients. No differences in incidence were detected according to age, sex, ethnicity or CCI (Table 1). Therefore, only overall changes in incidence are reported. In Table 2, Table 3 and Table 4, we present rates of STEMI, non-STEMI and CHF hospitalization per 1000 person-years for each period during the Corona year vs. the three baseline years.

**Figure 1:**
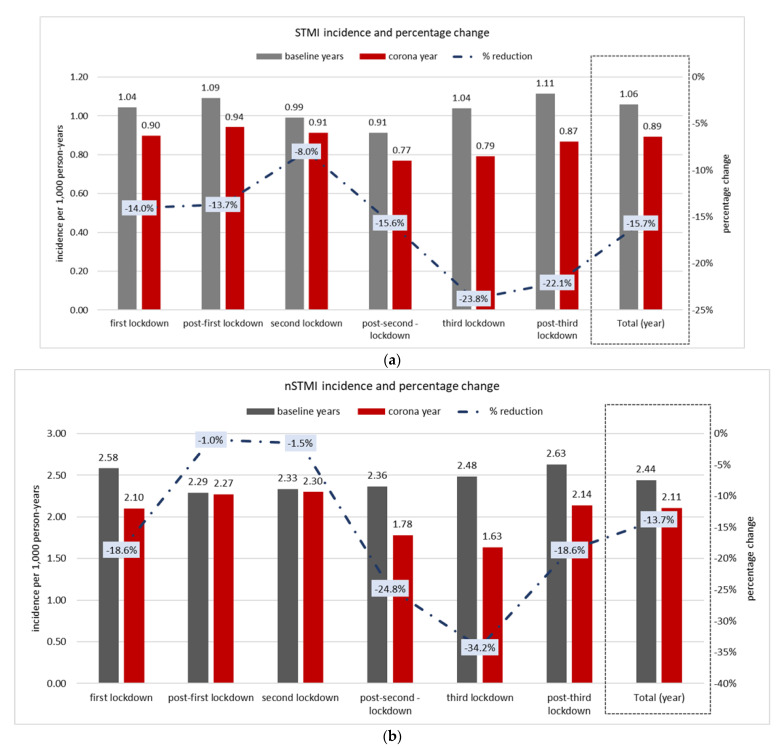

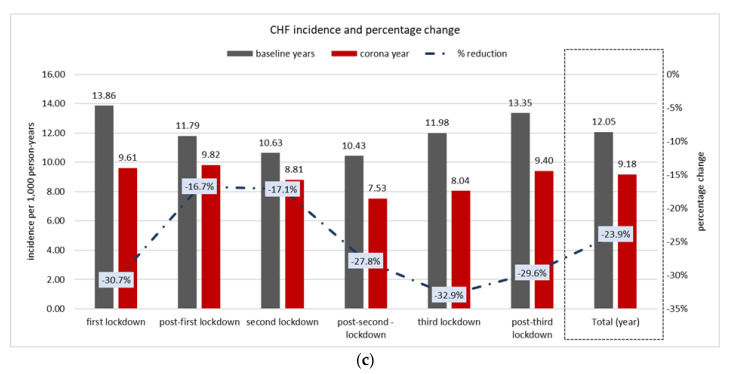
(**a**) STEMI incidence by period during the Corona year and the average of preceding three years, and percentage change (hospitalization cases per 1000 person-years). (**b**) Non-STEMI incidence by period during the Corona year and the average of preceding three years, and percentage change (hospitalization cases per 1000 person-years). (**c**) CHF incidence by period during the Corona year and the average of preceding three years, and percentage change (hospitalization cases per 1000 person-years).

The overall STEMI incidence rate during the Corona year was 0.89 cases per 1000 person-years, representing a decline of 15.7% (95% CI: 13%, 19%) relative to the baseline years. STEMI incidence rates were lower throughout the year and in particular during the third lockdown (24% decline relative to the baseline years; 95% CI: −21, −27%) and during the post-third lockdown period (22% decline relative to the baseline years; 95% CI: −19, −25%) (see Table 2 and Figure 1a). Non-STEMI incidence during the Corona year was 2.11 cases per 1000 person-years, representing a decline of 13.7% (95% CI: 11%, 17%) relative to the baseline years. In contrast to STEMI incidence, the decline in non-STEMI incidence was not significant in all of the periods: following a significant reduction in incidence of 19% relative to baseline during the first lockdown (95% CI: −16%, −22%), non-STEMI incidence rates rebounded to the vicinity of the baseline rates during the post-first lockdown period and the second lockdown period. Thereafter, incidence rates dropped by 25% in the post-second lockdown period (95% CI: −22%, −28%); by 34% in the third lockdown period (95% CI: −31%, −37%), and by 19% in the post-third lockdown period (95% CI: −16%, −22%) (see Table 3 and Figure 1b).

The overall CHF incidence during the Corona year was 9.18 cases per 1000 person-years, representing a decline of 23.9% (95% CI: −21%, −27%) relative to the baseline years. An examination of each period separately shows that the reduction in CHF incidence remained similar throughout the year, ranging from 17% in the post-first lockdown period and second lockdown period (95% CI: −14%, −20%) to a peak of 33% (95% CI: −30%, −36%) in the third lockdown period (see Table 4 and Figure 1c).

The increase in the CFR for the entire year was significant in the case of CHF (adjusted OR-adjOR 1.226 95% CI: 1.172–1.282) and borderline significant in the case of non-STEMI (adjOR 1.104 95% CI: 1.001–1.217), but was not significant in the case of STEMI (adjOR 1.091 95%CI: 0.934–1.275), although this varied from period to period.

**Figure 2:**
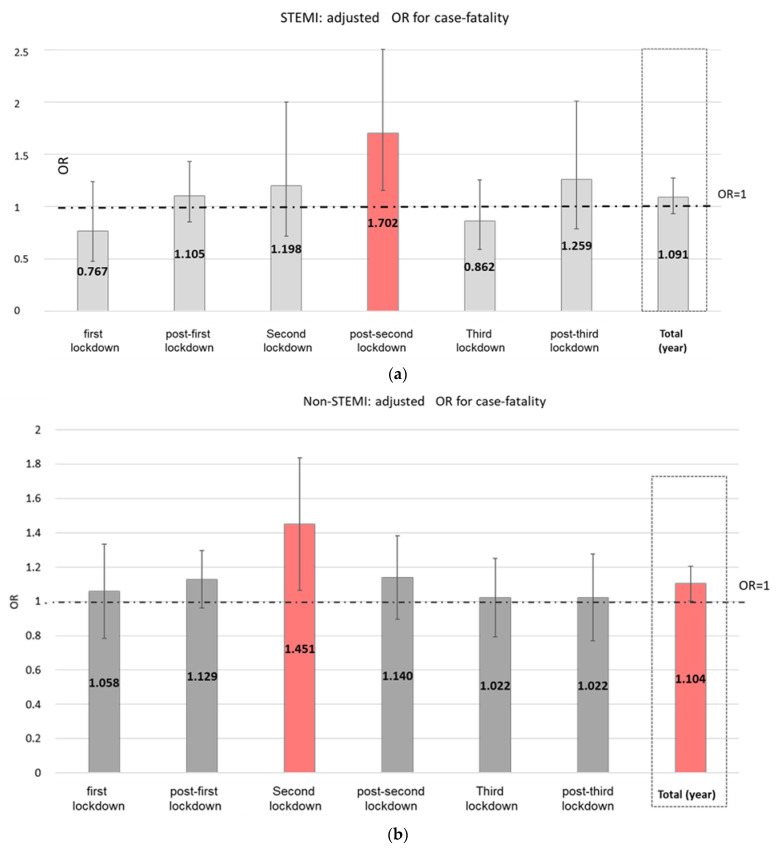

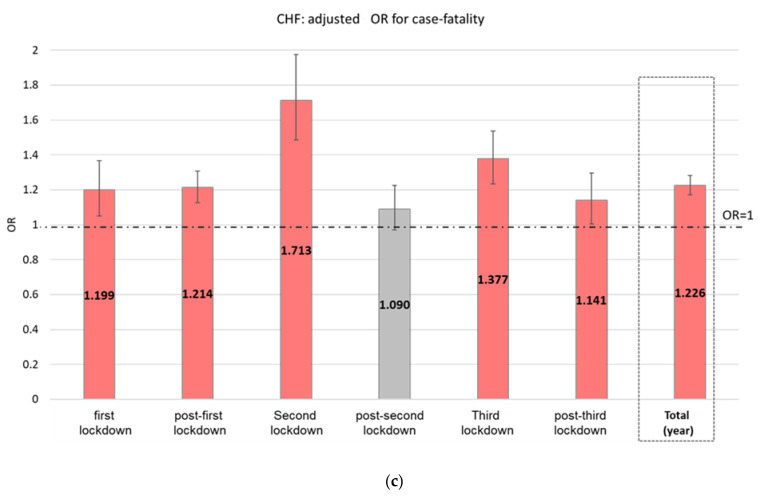
(**a**) STEMI adjusted case fatality rate during the Corona year vs. the corresponding baseline periods in the preceding three years (OR from GEE logistic models, adjusted for age, sex, and CCI). (**b**) Non-STEMI adjusted case fatality rate during the Corona year vs. the corresponding baseline periods in the preceding three years (OR from GEE logistic models, adjusted for age, sex, and CCI). (**c**) CHF adjusted case fatality rate during the Corona year vs. the corresponding baseline periods in the preceding three years (OR from GEE logistic models, adjusted for age, sex, and CCI).

An examination of each period separately shows a significant increase in the CFR among STEMI patients only during the post-second lockdown period (adjOR 1.702 95% CI: 1.155–2.508) (Figure 2a). In the case of non-STEMI patients, an increase in the CFR was observed only during the second lockdown period relative to the baseline years (adjOR 1.451 95%CI: 1.064–1.978) (Figure 2b). In contrast to the AMI patients, patients admitted with CHF during the Corona year had a higher CFR relative to the baseline years in all periods (except for the post-second lockdown period), reaching a peak during the second lockdown period (adjOR 1.713 95% CI: 1.486–1.974) (Figure 2c).

## 4. Discussion

The study showed a significant and continuous decline in AMI and CHF hospitalization rates during the first year of the COVID-19 pandemic in Israel relative to the previous three years: 15.7% in STEMI hospitalization (CI 95%: 13%, 19%), 13.7% in non-STEMI hospitalization (CI 95%: 11%, 17%) and 23.9% in CHF hospitalization (CI 95%: 21%, 27%). While 30-day all-cause mortality rates among both STEMI and non-STEMI patients during the Corona year were quite similar to those among AMI patients in the previous years, mortality rates among CHF patients were 22.6% higher (adjOR 1.226 95%CI: 1.172–1.282), and the differences were statistically significant for most of the study periods.

There are those who claim that an actual decrease in the incidence of cardiovascular events during the pandemic cannot be ruled out since staying at home may have reduced AMI [11,12] by limiting the exposure to external triggers of acute coronary events (such as pollution and workplace stress). However, the general consensus is that the indirect health effects of the pandemic reduced the consumption of cardiovascular hospitalization services [13]. Though the reasons for the decline are not fully understood, they are for the most part attributed to the imposition of social isolation, “shelter-in-place orders”, and similar regulations during the surges in COVID-19 cases. Their goal was to encourage the public not to visit medical centers and to avoid unnecessary healthcare use in order to reduce transmission of the virus and ensure that hospital capacity could accommodate surges in COVID-19 cases [14,15]. Certain patient populations also reported forgoing medical care, mainly owing to fear of SARS-CoV2 infection [16], or in an attempt to mitigate the burden on the healthcare system [17], in line with public health messages communicated by governments.

Various studies worldwide showed a different pattern of cardiovascular hospitalization rates later on in the pandemic. In the UK [8], a study showed similar declines in AMI and CHF hospitalizations during the first and second lockdown periods. The researchers suggest that the public were fearful of visiting hospitals and concluded that clear public messaging is necessary to prevent further unintended consequences of social distancing. Another study in the UK examined CHF hospitalization rates during the three COVID-19 surges and accompanying lockdowns and found that despite the public health messages and healthcare reconfiguration, the admission rates remained significantly reduced throughout the entire COVID-19 pandemic, with no difference between the three lockdown periods examined [18]. In contrast to these studies and in line with our findings, a study in Denmark [10] demonstrated a further reduction in cardiovascular hospitalization rates during the second COVID-19 surge, though it was smaller in magnitude than during the first lockdown. The researchers concluded that declines in cardiovascular admission rates may be preventable during COVID-19 case surges. A study carried out in the US [9] using data of Kaiser Permanente Northern California showed no significant decline in AMI hospitalization during the second lockdown, as compared to the decline observed in the first lockdown. According to the researchers, this may reflect changing patient attitudes during the COVID-19 pandemic or the success of public health campaigns to reassure patients that it is safe to seek emergency care when necessary [9].

The variability in the rates of decline in cardiovascular hospitalization rates across countries during the pandemic is evidence of the multiplicity of underlying factors. These include the non-uniform intensity of the pandemic across countries and variation in the stringency of lockdown measures. The variability may also be due to public-health-related factors, such as differences in the structure and infrastructure of the local health care system, its ability to provide a preventative community response and hospitalization substitute, and variation in both the messages conveyed to the public regarding the importance of coming to the ER during a medical emergency and the public’s level of trust. The variation in the rate of decline in cardiovascular hospitalization rates across the various surges shown in the Danish and American studies supports the existence of a reversible component of the decline, which can be minimized despite the surges in COVID-19 cases. Therefore, the decline in seeking medical attention may not be driven directly by lockdowns per se, but rather by other factors, such as fear of infection or lack of trust in the authorities [10].

According to our findings, the decline in the rate of cardiovascular hospitalizations weakened during the second surge (with non-STEMI hospitalizations returning to their normal level), but it reached a peak during the third surge. Therefore, and as demonstrated in other studies, the level of COVID-19 morbidity alone, which rose in Israel from the first surge to the second and from the second to the third, is not a sufficient explanation for this phenomenon. These findings highlight the importance of intensifying public health messaging and reinforcing the public’s confidence in the safety of visiting the hospital for emergency treatment in future COVID-19 surges, with the goal of mitigating the decline in cardiovascular hospitalization and the under-treatment of cardiovascular conditions.

As in other studies [18,19,20], we did not identify a significant rise in the 30-day all-cause mortality rates among AMI patients during most of the periods relative to previous years; however, we did find higher rates among CHF patients during almost all of the periods examined [21]. The shift of system resources to treatment of COVID-19 patients at the expense of providing optimal care to other patients is unlikely to explain our findings, in view of the fact that the burden of COVID-19 morbidity and the shift in system resources increased over the course of the year, while AMI and CHF mortality rates did not demonstrate a constant upward trend. Therefore, a possible explanation for our findings and for the difference in mortality rates between AMI patients and CHF patients may be the difference in the characteristics of patients arriving at the ER and in the severity of their condition. While AMI is an acute and urgent diagnosis, which usually has a clear presentation and results in a rapid evacuation to the ER and hospitalization, CHF often has a more moderate and gradual presentation and may be dealt with in the community and without an ER admission. Furthermore, as a result of these differences, some of the individuals suffering from CHF exacerbation may have delayed going to hospital and eventually arrived in a more severe condition due to their reluctance to be in a hospital environment [22]. Therefore, it is possible that while the severity of AMI among patients arriving at the hospital did not change significantly during the pandemic, CHF patients (whose number declined more than that of AMI patients) included a higher proportion of more critical patients who were characterized by a higher mortality rate. As found in other studies [21,23,24], we believe that cardiovascular patients who did arrive at the ER during the Corona year received care at a similar level to that in the preceding non-pandemic years and that the quality of care was not compromised during the pandemic. The increased rates of mortality among CHF patients may therefore reflect a change in mix of acuity among CHF patients arriving at the ER [18,25]. Similar findings of reduced CHF hospitalizations accompanied by an increase in 30-day all-cause mortality were reported in the UK [21] and in Germany [26]. Studies suggest that the quality of care for cardiovascular hospitalizations was not compromised, despite the structural and organizational changes during the COVID-19 pandemic, rather it was hospitalized patients who were sicker [21].

In conclusion, our findings show a continuous decline in cardiovascular hospitalizations during the Corona year and a possible change in the mix of CHF patients. This reinforces the need for public health officials to encourage patients not to delay necessary medical care during future COVID-19 surges and to initiate proactive long-term follow-up in order to identify the health consequences of the possible sub-optimal treatment of acute cardiovascular conditions during the Corona year.

This study has several strengths. First, we were able to examine weekly AMI and CHF hospitalization rates among members of a large integrative healthcare organization during the first year of the pandemic. Second, the length of the sample period, the extent of population coverage and the availability of up-to-date computerized data gathered in both inpatient and community settings provided a unique dataset of AMI and CHF hospitalizations. Finally, the accessibility and completeness of the data made it possible to examine 30-day all-cause mortality.

Limitations: The main limitation of the study is the lack of data for out-of-hospital cardiovascular mortality among individuals who refrained from going to the hospital. This limited our ability to fully analyze the pandemic’s potential collateral harm in discouraging individuals from seeking necessary treatment, even in emergencies. Furthermore, lack of data on concomitant COVID-19 infection in AMI and CHF patients may potentially affect the rates of cardiovascular hospitalization, in view of the possible increase in cardiac enzymes during a COVID-19 infection. The study is also limited to hospitals in Israel, and the results may not be generalizable to other countries.

## 5. Conclusions

We carried out a large retrospective cohort study which demonstrated a significant reduction in AMI and CHF hospitalizations during the first year of the pandemic in Israel, relative to the preceding three years. Although 30-day all-cause mortality did not change significantly in the case of AMI patients, it increased among CHF patients hospitalized during the first year of the pandemic, possibly due to a worsening in the clinical acuity of patients arriving at the hospitals, rather than lower standards of care. The study’s findings have a number of implications for future surges in COVID-19 morbidity, as follows: Practitioners and public health officials should encourage patients not to delay essential medical care that cannot be managed in other settings [15], in order for the public to understand the importance of seeking guidance and emergency care for acute cardiovascular conditions. Furthermore, possible barriers to the provision of necessary medical care should be addressed by means of targeted public health messaging, with the goal of preventing collateral cardiovascular damage [27]. Further research is needed in order to proactively monitor the potential consequences over time of forgoing or deferring care for acute cardiovascular conditions during the Corona year.

## Figures and Tables

**Table 1:** Population and patient characteristics, Corona year and average of three baseline years.

		Population			STEMI			non-STEMI			CHF		
		Yearly Average of 3 Baseline Years	Corona Year	*p* Value	Yearly Average of 3 Baseline Years	Corona Year	*p* Value	Yearly Average of 3 Baseline Years	Corona Year	*p* Value	Yearly Average of 3 Baseline Years	Corona Year	*p* Value
**Total**	N	4,494,077	4,627,464		3187.3	2758		7353.3	6509		36,285	28,352	
Age	Age < 60 years	3,675,782 (82%)	3,782,214 (82%)	0.024	1089 (34%)	974 (35%)	NS	1482 (20%)	1315 (20%)	NS	3098 (9%)	2377 (8%)	NS
	>=60 years	818,295 (18%)	845,250 (18%)		2098 (66%)	1784 (65%)		5872 (80%)	5194 (80%)		33,187 (91%)	25,975 (92%)	
Sex	Male	2,204,749 (49%)	2,274,502 (49%)	0.005	2430 (76%)	2117 (77%)	NS	4929 (67%)	4439 (68%)	NS	19,324 (53%)	15,184 (54%)	NS
	Female	2,289,313 (51%)	2,352,914 (51%)		757 (24%)	641 (23%)		2424 (33%)	2070 (32%)		16,962 (47%)	13,168 (46%)	
Charlson comorbidy index	CCI < 5	4,354,719 (97%)	4,481,813 (97%)	<0.001	823 (26%)	700 (25%)	NS	1584 (22%)	1312 (20%)	NS	14,071 (39%)	11,167 (39%)	NS
	CCI >= 5	139,358 (3%)	145,651 (3%)		69 (2%)	59 (2%)		200 (3%)	166 (3%)		2221 (61%)	17,185 (61%)	
Ethnicity	Arab	1,210,338 (27%)	1,248,746 (27%)	<0.001	2295 (72%)	1999 (72%)	NS	5568 (76%)	5031 (77%)	NS	7255 (20%)	5546 (20%)	NS
	Ultra-Orthodox	253,545 (6%)	273,004 (6%)		2532 (79%)	2233 (81%)		4557 (62%)	4071 (63%)		1136 (3%)	891 (3%)	
	General	3,013,349 (67%)	3,090,087 (67%)		655 (21%)	525 (19%)		2797 (38%)	2438 (37%)		27,885 (77%)	21,913 (77%)	
	Missing	16,845 (0%)	15,627 (0%)										

**Table 2:** STEMI incidence by period for the Corona year and the baseline years.

STEMI		First Lockdown	Post-First Lockdown	Second Lockdown	Post-Second -Lockdown	Third Lockdown	Post-Third Lockdown	Total
	Calendar weeks	10 to 15	16 to 36	37 to 41	42 to 51	52 to 6	7 to 10	
Baseline years	N of cases	1068	3905	845	1556	1239	949	9562
	Total person-years	1,022,379	3,578,329	851,983	1,703,966	1,192,777	851,983	9,031,021
	Incidence per 1000 PY	1.04	1.09	0.99	0.91	1.04	1.11	1.06
Corona year	N of cases	314	1153	266	449	323	253	2758
	Total person-years	349,688	1223,909	291,407	582,814	407,970	291,407	3,088,913
	Incidence per 1000 PY	0.90	0.94	0.91	0.77	0.79	0.87	0.89
**% reduction**		**−14%**	**−13.7%**	**−8%**	**−15.6%**	**−23.8%**	**−22%**	**−15.7%**
95% CI (upper, lower)		−11%, −17%	−11%, −17%	−5%, −11%	−13%, −19%	−21%, −27%	−19%, −25%	−13%, −19%

**Table 3:** Non-STEMI incidence by period during the Corona year and baseline years.

Non-STEMI		First Lockdown	Post-First Lockdown	Second Lockdown	Post-Second-Lockdown	Third Lockdown	Post-Third Lockdown	Total
	Calendar weeks	10 to 15	16 to 36	37 to 41	42 to 51	52 to 6	7 to 10	
Baseline years	N of cases	2640	8201	1989	4027	2964	2239	22,060
	Total person-years	1,022,379	3,578,329	851,983	1,703,966	1,192,777	851,983	9,031,021
	Incidence per 1000 PY	2.58	2.29	2.33	2.36	2.48	2.63	2.44
Corona year	N of cases	735	2778	670	1036	667	623	6509
	Total person-years	349,688	1,223,909	291,407	582,814	407,970	291,407	3,088,913
	Incidence per 1000 PY	2.10	2.27	2.30	1.78	1.63	2.14	2.11
	**% reduction**	**−18.6%**	**−1%**	**−1.5%**	**−24.8%**	**−34.2%**	**−18.6%**	**−13.7%**
	95% CI (upper, lower)	−16%, −22%	2%, −4%	1%, −5%	−22%, −28%	−31%, −37%	−16%, −22%	−11%, −17%

**Table 4:** CHF incidence by period during the Corona year and baseline years.

CHF		First Lockdown	Post-First Lockdown	Second Lockdown	Post-Second–Lockdown	Third Lockdown	Post-Third Lockdown	Total (Year)
	calendar weeks	10 to 15	16 to 36	37 to 41	42 to 51	52 to 6	7 to 10	
Baseline years	N of cases	14,167	42,191	9058	17,771	14,295	11,374	108,856
	Total person-years	1,022,379	3,578,329	8,51,983	1,703,966	1,192,777	851,983	9,031,021
	Incidence per 1000 PY	13.86	11.79	10.63	10.43	11.98	13.35	12.05
Corona year	N of cases	3359	12018	2568	4387	3281	2739	28352
	Total person-years	349,688	1,223,909	291,407	582,814	407,970	291,407	3,088,913
	Incidence per 1000 PY	9.61	9.82	8.81	7.53	8.04	9.40	9.18
**% reduction**		**−30.7%**	**−16.7%**	**−17.1%**	**−27.8%**	**−32.9%**	**−29.6%**	**−23.9%**
95% CI (upper, lower)		−28%, −34%	−14%, −20%	−14%, −20%	−25%, −31%	−30%, −36%	−27%, −33%	−21%, −27%

## Data Availability

Data are available upon request.
